# Experimental Implementation of a Biometric Laser Synaptic Sensor

**DOI:** 10.3390/s131217322

**Published:** 2013-12-16

**Authors:** Alexander N. Pisarchik, Ricardo Sevilla-Escoboza, Rider Jaimes-Reátegui, Guillermo Huerta-Cuellar, J. Hugo García-Lopez, Victor B. Kazantsev

**Affiliations:** 1 Centro de Investigaciones en Optica, Loma del Bosque 115, Lomas del Campestre, Leon 37150, Guanajuato, Mexico; 2 Centre for Biomedical Technology, Technical University of Madrid, Campus Montegancedo, Pozuelo de Alarcon 28223, Madrid, Spain; 3 Centro Universitario de Los Lagos, Universidad de Guadalajara, Enrique Díaz de León 1144, Paseo de la Montaña, Lagos de Moreno, Jalisco 47460, Mexico;E-Mails: sevillaescoboza@gmail.com (R.S.-E.); rjaimes@culagos.udg.mx (R.J.-R.); guillermo.huerta@ipicyt.edu.mx (G.H.-C); jhgarcial@yahoo.com (J.H.G.-L.); 4 Institute of Applied Physics of Russian Academy of Science, 46 Uljanov Str., Nizhny Novgorod 603950, Russia; E-Mail: kazantsev@neuro.nnov.ru; 5 Lobachevsky State University of Nizhni Novgorod, 23 Gagarin Ave., Nizhny Novgorod 603950, Russia

**Keywords:** fiber laser, electronic circuit, neuron, synapse, artificial intelligence, neuroengineering, biorobotics, pump modulation

## Abstract

We fabricate a biometric laser fiber synaptic sensor to transmit information from one neuron cell to the other by an optical way. The optical synapse is constructed on the base of an erbium-doped fiber laser, whose pumped diode current is driven by a pre-synaptic FitzHugh–Nagumo electronic neuron, and the laser output controls a post-synaptic FitzHugh–Nagumo electronic neuron. The implemented laser synapse displays very rich dynamics, including fixed points, periodic orbits with different frequency-locking ratios and chaos. These regimes can be beneficial for efficient biorobotics, where behavioral flexibility subserved by synaptic connectivity is a challenge.

## 1. Introduction

The human brain consists of approximately one hundred billion neurons interconnected in a complex way to perform computational, cognition and memory tasks. Neurons usually interact via synapses, which allow information transmission from one cell to the other. Each neuron has an average of 7,000 synaptic connections. Being subserved by a complex molecular mechanism, the synapses are capable of changing the efficiency of signal transmission between neurons by sensing electrical activity and chemical concentrations. It is believed that the flexibility of the synaptic connections (e.g., synaptic plasticity) underlies the implementation of computational and cognitive tasks in brain networks. While in living cells, the synaptic plasticity is mediated by complex molecular transformation, in an artificial biosystem, the synaptic transmission can be regulated by adjusting the parameters of an artificial synapse.

Significant efforts have been made to represent a synapse by a single device that mimics synaptic connections and behavioral flexibility. This challenging task for biorobotics would allow a direct linkage between neuroscience and artificial intelligence. Any attempt to construct an artificial brain must consider its complexity. Several attempts have already been made. For example, Sharp *et al.* [1] used an electronic circuit to couple two living stomatogastric ganglia neurons. Synaptic behavior has also been imitated by hardware-based neural networks, such as hybrid complementary metal-oxide-semiconductor analogue circuits and other artificial neural devices [2–4] capable of mimicking the major features of human memory; namely, sensory, short-term and long-term memories. Great progress in nanotechnology has provided significant advances in the miniaturization of synthetic synapses, e.g., the fabrication of a carbon nanotube synaptic circuit [5]. Artificial synaptic devices based on ion migration have been also designed [6]; some of them [7,8] have demonstrated spike-timing-dependent plasticity [9], the important mechanism of brain memory, related to the synaptic connection strength in biological circuits and synthetic devices. These devices require precise control of the signal timing to simulate the pre- and post-synaptic potentials in biological systems.

Another approach in constructing a synthetic brain is to make it completely artificial without linking to time and space scales of real neurons, in other words, to connect artificial neurons by artificial synapses. This approach has several advantages over traditional approaches. First, such a device does not require very strong miniaturization, and second, the time scale can be much shorter (*i.e.*, a much higher spike frequency) than in real neurons. The fast oscillation frequency of electronic neurons in comparison with real neurons can be beneficial for an artificial biosystem to increase the speed of its activity. Several neuron models have been developed to mimic biological neuron dynamics. The simplified modification of the detailed Hodgkin-Huxley model [10], the FitzHugh–Nagumo (FHN) model [11], has attracted much attention, because of its easy implementation as an analog electronic circuit, which simulates spike-timing neural activity [12–14].

Recently, we have proposed an optical synaptic sensor based on an erbium-doped fiber laser (EDFL) to establish a functional connection between FHN electronic neurons [15]. We have numerically shown that this laser synapse allows one to control the spike transmission with a very high flexibility. The distinguished features of the laser synapse from other artificial (electronic) synapses are: (1) an optical carrier (optical radiation in the IRspectral range) instead of an electric current; (2) optical fiber transmission instead of a metallic wire; and (3) very rich dynamics, including fixed points, periodic orbits with different frequency-locking ratios and chaos.

In this work, we report on the first, to the best of our knowledge, experimental implementation of the laser synapse and demonstrate its high flexibility in controlling signal transmission from a pre-synaptic to a post-synaptic neuron.

## 2. Experimental Setup

**Figure 1.**
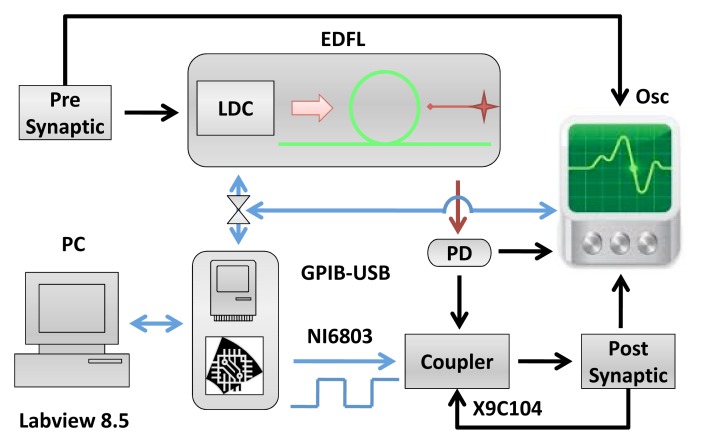
Experimental setup. The pre-synaptic FitzHugh–Nagumo (FHN) electronic circuit drives the erbium–doped fiber laser (EDFL) via the laser diode controller (LDC). The signal from the photodetector (PD) after passing through the coupler controls the post-synaptic FHN electronic circuit. The output signals from the pre-synaptic neuron, laser and post-synaptic neuron are recorded by the oscilloscope (Osc) and stored in the computer (PC) using the GPIBcommunication protocol and Labview 8.5. The square signal generated by the NI6803 card is applied to the digital potentiometer, X9C104, to control the coupling strength.

The experimental setup is shown in Figure 1. The EDFL is pumped by a laser diode (wavelength: 976 nm) through a wavelength-division multiplexing coupler and a polarization controller. The laser cavity of a 1.55-m length is formed by a piece of erbium-doped fiber of 70 cm in length and 2.7 *μ*m in core diameter and two fiber Bragg gratings with a 2-nm FWHMbandwidth and with 90.5% and 94% reflectivity at a 1,550-nm wavelength. The diode pump laser is controlled by a laser diode controller (LDC)(Thorlabs ITC510).

**Figure 2.**
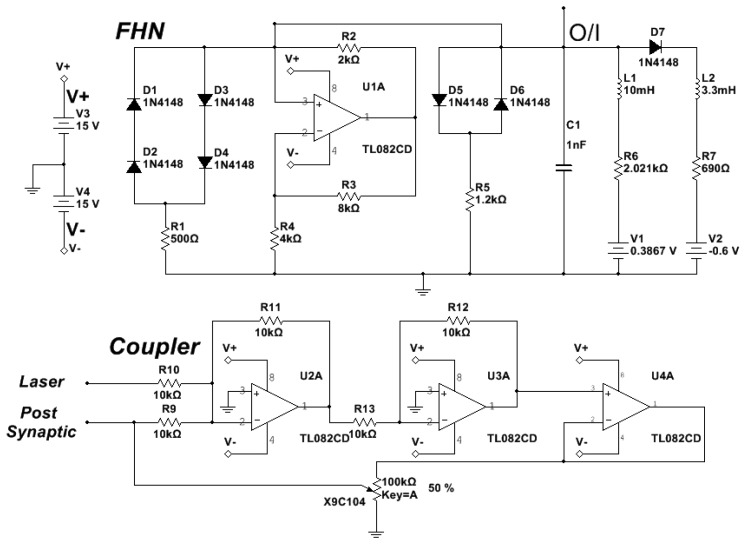
Electronic schemes of FitzHugh–Nagumo and coupler circuits.

The diode pump current of the EDFL is modulated by the pre-synaptic neuron. The optical output of the EDFL is converted to an electrical signal by the photo-detector and sent through the coupler to the post-synaptic neuron. Figure 2 shows the electronic schemes of the FHN circuit and coupler [13,14].

**Figure 3.**
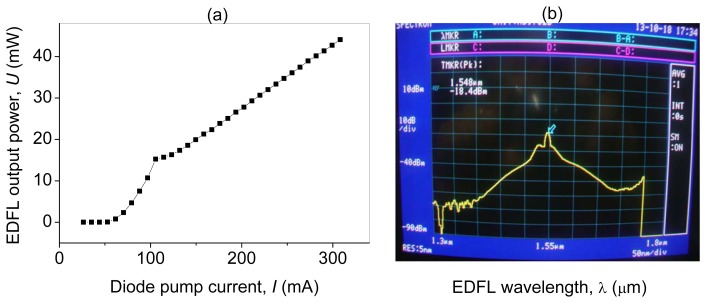
**(a)** EDFL output power *versus* diode pump current and (**b**) the EDFL optical spectrum.

The EDFL output power depends linearly on the diode pump current, *I*, as shown in Figure 3a. The lasing threshold is 107 mA. Figure 3b shows the optical spectrum of the EDFL.

## 3. Results and Discussion

### 3.1. Synaptic Transfer Function

**Figure 4.**
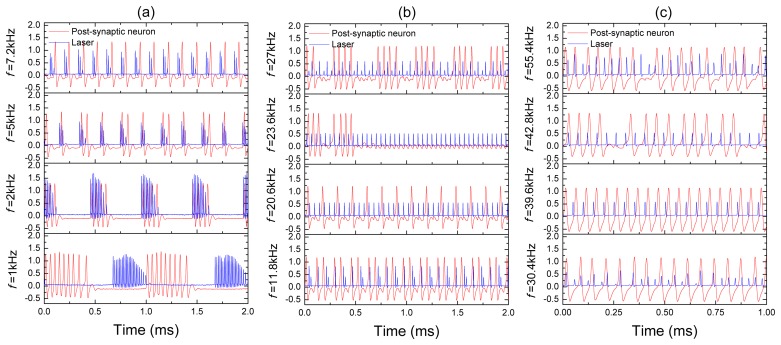
Laser (blue traces) and post-synaptic neuron (red traces) responses to harmonic modulation at (**a**) low, (**b**) middle and (**c**) high frequencies. The amplitude of the input signal applied to the laser pump current from a signal generator *A* = 1 V, and *I* = 125 mA.

Before connecting the pre-synaptic neuron to the post-synaptic neuron, we measure a transfer function of the laser synapse. The transfer function is a frequency response of the synapse to an input signal. This important characteristic provides us with information about the frequency resolution of the synaptic sensor, *i.e.*, its sensitivity to input frequency. Figure 4 shows the frequency resolution of the laser synapse (blue traces) and the post-synaptic neuron (red traces) to a harmonic signal applied to the laser pump current. The input frequency is indicated on the left-hand side of each time series.

**Figure 5.**
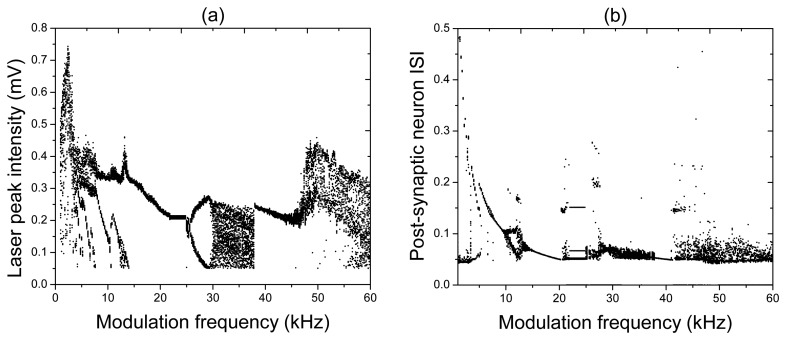
Bifurcation diagrams of (**a**) laser peak intensity and (**b**) post-synaptic neuron inter-spike-interval (ISI) using modulation frequency as a control parameter. *A* = 1 V, and *I* = 125 mA.

For very low input frequencies (Figure 4a), a train of the laser and post-synaptic neuron spikes emerges at every period of the input signal. Inside each train of pulses, the synapse and post-synaptic neuron respond at different frequencies, and the number of spikes in the train decreases as the input frequency is increased. At higher frequencies (Figure 4b), it can happen that the post-synaptic neuron either stays silent (for *f* = 23.6 kHz), or there is a spike train (for *f* = 20.5 kHz and / = 27 kHz) regime while the laser emits a pulse at every period of the input signal. For high frequencies (Figure 4c), the response of the post-synaptic neuron to a chaotic laser input can be either periodic (for *f* = 30.4 kHz) or irregular (for *f* = 55.4 kHz). All these and other regimes can be distinguished in the bifurcation diagrams of the laser peak intensity and the post-synaptic neuron inter-spike-interval (ISI) shown, respectively, in Figure 5a,b.

While the bifurcation diagram of the laser peak intensity is the transfer function of the laser synaptic sensor, the ISI is the transfer function of the system formed by the laser and the post-synaptic neuron. The diversity of dynamical regimes obtained in the laser and its high sensitivity to the input frequency indicate a high flexibility of the laser synapse that can be beneficial for controlling signal transmission from one neuron to the other.

### 3.2. Neuron Connection

**Figure 6.**
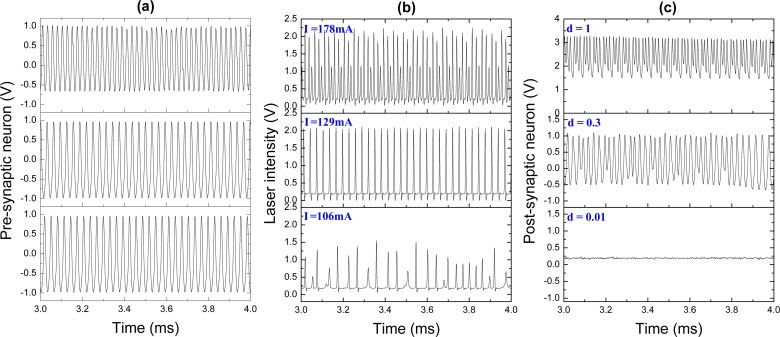
Time series of the (**a**) pre-synaptic neuron, (**b**) laser and (**c**) post-synaptic neuron. The laser response in (b) displays the 2:3 (upper trace) and 1:1 (middle trace) frequency-locking and chaotic (lower trace) regimes, while the post-synaptic neuron response in (c) exhibits the 2:3 (upper trace), unlocked (middle trace) and silence (lower trace) regimes.

We now consider the artificial neuron system formed by pre- and post-synaptic electronic neurons connected by the laser synapse. In the previous subsection, we showed that the ability of our laser synaptic sensor to transmit information from one neuron to the other is determined by the neuron spike frequency. Our pre-synaptic FHN electronic circuit generates periodic spikes with a frequency of 32 kHz (Figure 6a). These pulses serve as an input signal to control the diode pump current. The laser response to this input is shown in Figure 6b. Different pump currents *I* result in different laser waveforms, from periodic (upper and middle traces) to chaotic (lower trace). The response of the post-synaptic neuron to the laser input, *i.e.*, the response to the signal from the pre-synaptic neuron transmitted through the laser synapse, is shown in Figure 6c. To control the dynamics of the post-synaptic neuron, we can manipulate by the synaptic strength, *d*, which acts as a coupling coefficient between the laser and the post-synaptic neuron. The spike trains in Figure 6c for different coupling strengths are obtained for the laser input signals at the corresponding rows in Figure 6b.

**Figure 7.**
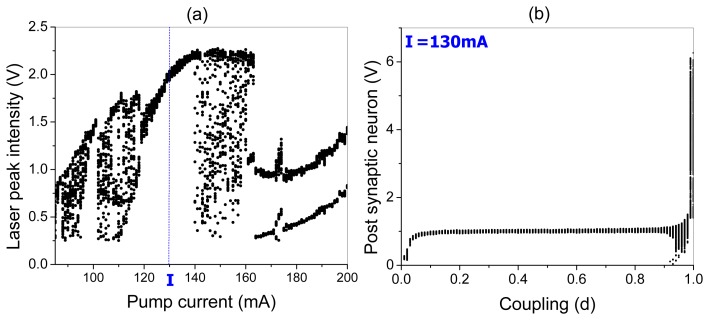
Bifurcation diagrams of (**a**) laser peak intensity and (**b**) post-synaptic neuron peak potential.

The bifurcation diagrams of the laser peak intensity *versus* the pump current and the peak potential of the post-synaptic neuron *versus* the coupling strength are shown in Figure 7a,b, respectively. One can see that EDFL modulated by the pre-synaptic neuron displays a very rich dynamics. For small pump currents, the laser generates chaotic spikes. When the pump current is increased from 115 mA to 140 mA, the laser oscillates periodically with the same frequency as the frequency of the pre-synaptic neuron spikes (1:1 frequency locking). For higher pump currents (from 140 mA to 160 mA), the laser again behaves chaotically, and for strong currents, the laser oscillations are locked as 2:1 or 3:1.

In the following, we fix the pump current at 130 mA corresponding to 1:1 frequency locking between the laser and pre-synaptic neuron, and measure the response of the post-synaptic neuron. As seen from the bifurcation diagram in Figure 5b, the post-synaptic neuron peak potential increases for small coupling *(d* < 0.1) and remains almost the same for intermediate coupling strengths (0.1 < *d* < 0.9), whereas for strong coupling *(d* > 0.9), it highly increases. Although, for medium couplings, the peak potential of the post-synaptic neuron is almost constant and approximately equal to 1 V, the spike frequency changes to different winding numbers *p : q (p* and *q* being integers).

### 3.3. Frequency Diagrams

**Figure 8.**
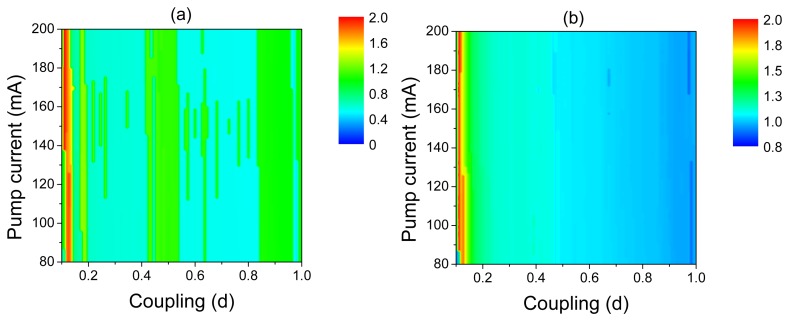
Frequency diagrams of the post-synaptic neuron in the space of coupling strength and laser pump current. The color indicates the ratio between the main oscillation frequency of (a) post-synaptic neuron spikes and laser pulses and (b) post-synaptic and pre-synaptic neuron spikes. The regions of different dynamical regimes correspond to different winding numbers *p : q*.

Different frequency-locking regimes can be distinguished in the frequency diagrams of the post-synaptic neuron in the space of the control parameters, *d* and *I*, shown in Figure 8. The Arnold tongue structure of these diagrams reflects the frequency-locking phenomenon. For very small coupling *(d* < 0.02), the post-synaptic neuron stays in a silent regime, *i.e.*, in the regime of subthreshold oscillations. For stronger coupling, the post-synaptic neuron fires with different frequency ratios with respect to oscillations of the laser and pre-synaptic neuron. Finally, for very strong coupling, the 1:1 frequency-locking regime is observed.

## 4. Conclusions

We implemented a biometric laser fiber synaptic sensor based on an erbium-doped fiber laser, whose pump current is controlled by a FitzHugh–Nagumo electronic circuit. We demonstrated its flexibility and functionality to transmit information from a pre-synaptic to a post-synaptic neuron. Depending on the diode pump current and the coupling strength between the laser and post-synaptic neuron, their outputs display different dynamical regimes, including silence, periodic frequency-locking regimes and chaos. We believe that the rich dynamics of the laser synapse can be very promising for biorobotic and neuroengineering applications, due to its relative simplicity and high flexibility, and may solve important problems in neuroengineering, such as, reproducing neuron dynamics and enhancing the functionality of neuromimetic networks. Further miniaturization of the FHN electronic circuits and opto-electronic components, e.g., in the form of microchips, would make such applications more efficient. The idea of the fiber synaptic sensor can also be interesting for neuroscientists to monitor and manipulate neuron functions via the optoelectronic interface between living and artificial neurons.
